# Supplementary material for: Can One Series of Self-Organized Nanoripples Guide Another Series of Self-Organized Nanoripples during Ion Bombardment: From the Perspective of Power Spectral Density Entropy?
Source: Entropy (Basel). 2023 Jan 14;25(1):170. doi: 10.3390/e25010170 (PMC9857890; doi:10.3390/e25010170)
Supplement: Supplementary file 1 [file entropy-25-00170-s001.zip › entropy-2028466-supplementary.pdf]

Supplement for supporting content:

Can one series of self-organized nanoripples guide another series of self-organized nanoripples during ion bombardment: from the perspective of power spectral density entropy?

Hengbo Li[1], Jinyu Li[1], Gaoyuan Yang[1], Ying Liu[1 *], Frank Frost[2], Yilin Hong[1]

1 National Synchrotron Radiation Laboratory, University of Science and Technology of China,

Hezuohua South Road 42, 230029 Hefei, Anhui, P. R. China

2 Leibniz Institute of Surface Engineering (IOM), Permoserstraße 15, 04318 Leipzig, Germany

* *Corresponding author:* liuychch@ustc.edu.cn

Tables S1–S4 show the main irradiation conditions of ARC, PR, PR/ARC and Au/ARC at different stages.

Figures S1–S4 show the PSD plots in logarithmic scales of the AFM images of Figures 1(a–e), Figures 2(a–e), Figures 3(a–e) and Figures 4(a–e).

In this study, the area of 5 μm × 5 μm of an AFM image is sufficient large for PSD entropy analysis. Figure S5 shows the original and normalized PSD entropies of the nanoripples on the irradiated Au/ARC surfaces calculated from the raw PSD data of the corresponding AFM with sizes of 5 μm × 5 μm (in red), 2.5 μm × 2.5 μm (in green), and 1.25 μm × 1.25 μm (in blue). The pixels of the AFM images are 1024×1024, 512×512, and 256×256. The average wavelength of the nanoripples is approximately 100 nm, as shown in Figures 7(c–e). The temporal evolutions of the PSD entropy for the AFM images with different image sizes exhibit a similar trend. Moreover, with increasing AFM size, the PSD entropy of the nanoripples increases, and the corresponding error bar of the PSD entropy decreases. Although the PSD entropy for the 1.25 μm ×1.25 μm AFM image appears as the lowest curve, the corresponding error bar of the curve shows the maximal fluctuation. As shown in Figure S5(b), the

normalized PSD entropy curves calculated from the AFM images with sizes of 5 μm × 5 μm (in red) and 2.5 μm × 2.5 μm (in green) almost overlap each other.

For patterns with the same lateral periodicity, we simulate the dependence of the PSD curves and entropies on the height fluctuation under several conditions. In the simulation, to conveniently tune pattern profiles with the same or similar lateral periodicity, i.e., same or similar PSD curves, we use rectangular diffraction gratings with a period of 78.125 nm and a fill factor of 0.5. The fill factor is defined as the ratio of the grating ridge width to its period. The grating period of 78.125 nm is determined by setting 64 periods within a 5 μm scanning length. There are 16 pixels for one grating period. This may lead to 1024×1024 pixels for a 5 μm ×5 μm grating image, similar to an AFM image of nanoripples. The height fluctuation is set with the function rand() in MATLAB software.

First, for nanoripples, the PSD entropy of nanoripples with uniform height and the same lateral periodicity does not depend on the scanning length or size of grating patterns and absolute ripple height (Figures S6(a–d)). The PSD entropy of gratings with different absolute ripple heights may shift up or down the amplitude of the evaluated PSD function, which can be deduced from the simulation data shown in Figures S6(c) and (d). Figures S6(c) and (d) show the PSD curves and PSD entropies of gratings with uniform heights of 100 nm, 200 nm and 300 nm. The absolute value of the intensity of a PSD curve depends on grating height. The higher the grating height is, the larger the amplitude of the PSD curve. Note that the normalized PSD curves of these gratings with different heights transform into identical curves. This also indicates that all these gratings have the same lateral periodicity. Correspondingly, the PSD entropies of all these gratings are equal to each other (Figure S6(d)). This agrees well with the fact that PSD curves are used to characterize the lateral periodicity of patterns.

Second, the PSD entropies are vulnerable to the height fluctuation of gratings with a specific average height, as shown in Figure S7(b); the larger the height fluctuations are, the higher the PSD entropy is. In fact, the amplitudes of the PSD curves of these gratings near the base frequency are almost identical [orange line in Figure S7(a)]. The

amplitudes of the PSD curves abruptly decrease at least four magnitudes less than that of the base frequency as the frequency deviates from the base frequency.

Figure S8 shows the temporal evolution of the lateral correlation length of each sample. The lateral correlation length is proportional to the inverse of the PSD entropy, meaning that the better the degree of ordering of the nanoripples is, the larger the lateral correlation length, and the lower the PSD entropy. Comparing with the lateral correlation length, we summarized the advantage of PSD entropy:

(1) The calculation of PSD entropy does not depend on the shape of the PSD curve. PSD entropy can be calculated for PSD curves without an imperfect PSD peak or even no clear peak, e.g., the PSD curves of the morphologies at stages A and B of the Au/ARC sample (Figures 4(a') and (b')). However, the lateral correlation length can only be calculated when the corresponding PSD curve shows an obvious high-frequency peak.

(2) In general, for a nanorippled morphology, the value of the error bar of its PSD entropy is less than that of the corresponding lateral correlation length.

Table S1 Irradiation conditions of the ARC sample at different stages.

Ion energy	Angle	Stage	Time (min)	Ion fluence (ions/cm^2)
400 eV	50°	Initial		
		A	2	1.8×10^{17}
		B	5	4.5×10^{17}
		C	7	6.3×10^{17}
		D	9	8.1×10^{17}
		E	11	9.9×10^{17}

Table S2 Details of the irradiated PR and the corresponding irradiation conditions at different stages.

Ion energy	Angle	Stage	Time (min)	Ion fluence (ions/cm^2)
400 eV	50°	Initial		
		A	6	5.6×10^{17}
		B	10	9.0×10^{17}
		C	20	1.8×10^{18}
		D	30	2.7×10^{18}
		E	40	3.6×10^{18}

Table S3 Details of the irradiated PR/ARC bilayer and the corresponding irradiation conditions at different stages.

Irradiated layer	Ion energy	Angle	Stage	Time (min)	Ion fluence (ions/cm^2)
			Initial		
PR			A	16	1.4×10^{18}
ARC	400 eV	50°	B	18	1.6×10^{18}
ARC			C	20	1.8×10^{18}
ARC			D	22	2.0×10^{18}
ARC			E	24	2.2×10^{18}

Table S4 Details of the irradiated Au/ARC bilayer and the corresponding irradiation conditions at different stages.

Thickness (nm) or position of the bilayer	Ion energy	Angle	Stage	Time (min)	Ion fluence (ions/cm^2)
~200(Au)/430(ARC)			Initial		
Au		80°	A	20	1.8×10^{18}
ARC, near the interface of Au and ARC	400 eV		B	22	2.0×10^{18}
		Sample rotated by 90° to keep the direction of the ripple vector of Au parallel to that of ARC			
ARC			C	26.5	2.4×10^{18}
ARC		50°	D	31.5	2.8×10^{18}
ARC			E	37.5	3.4×10^{18}

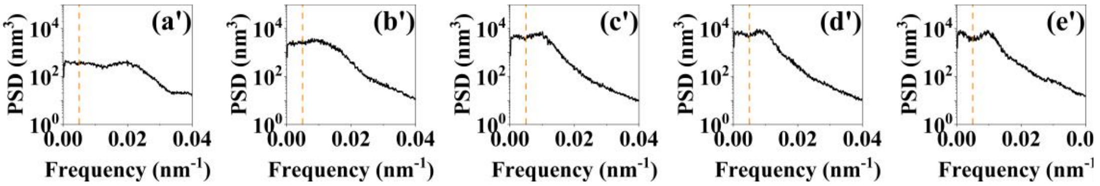

Figure S1. (a'–e') PSD curves in logarithmic scales of the AFM images shown in Figures 1(a–e).

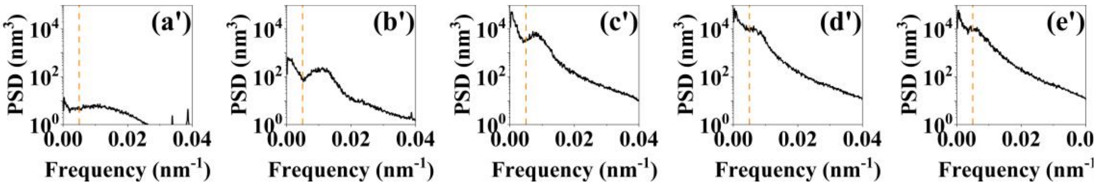

Figure S2. (a'–e') PSD curves in logarithmic scales of the AFM images shown in Figures 2(a–e).

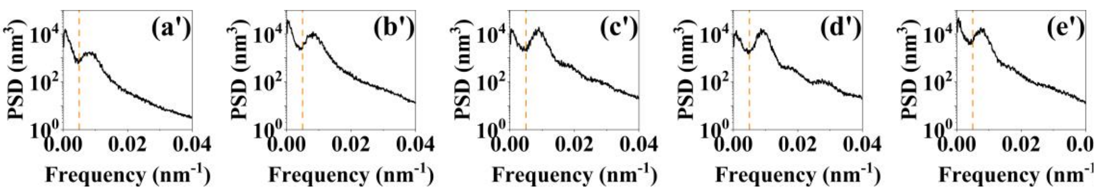

Figure S3. (a’–e’) PSD curves in logarithmic scales of the AFM images shown in Figures 3(a–e).

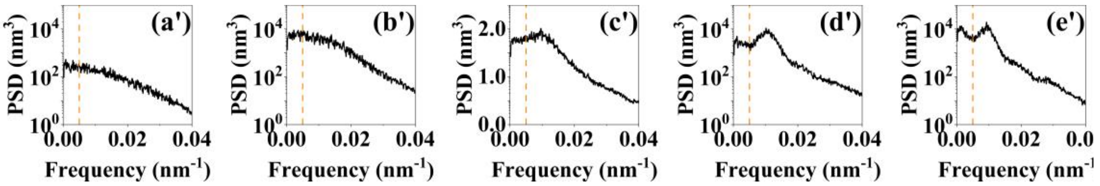

Figure S4. (a’–e’) PSD curves in logarithmic scales of the AFM images shown in Figures 4(a–e).

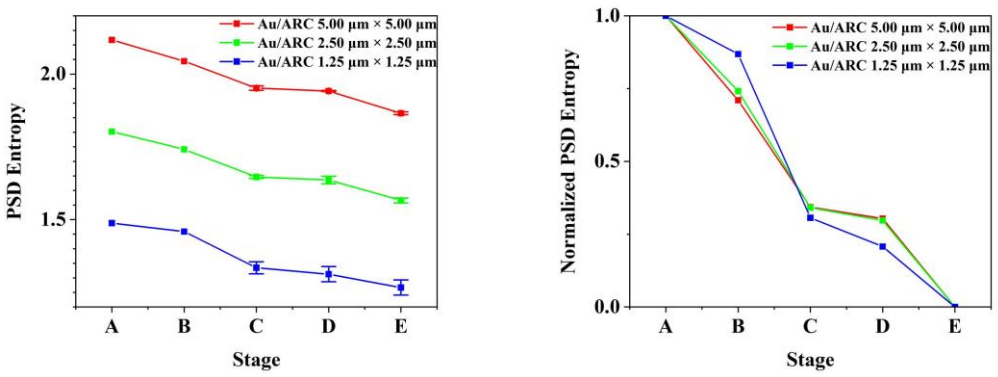

(a) (b)

Figure S5 (a) Original and (b) normalized PSD entropy of the nanoripples on the irradiated Au/ARC surfaces calculated from the raw PSD data of the corresponding AFM images with sizes of 5 μm × 5 μm (in red), 2.5 μm ×2.5 μm (in green), and 1.25 μm ×1.25 μm (in blue).

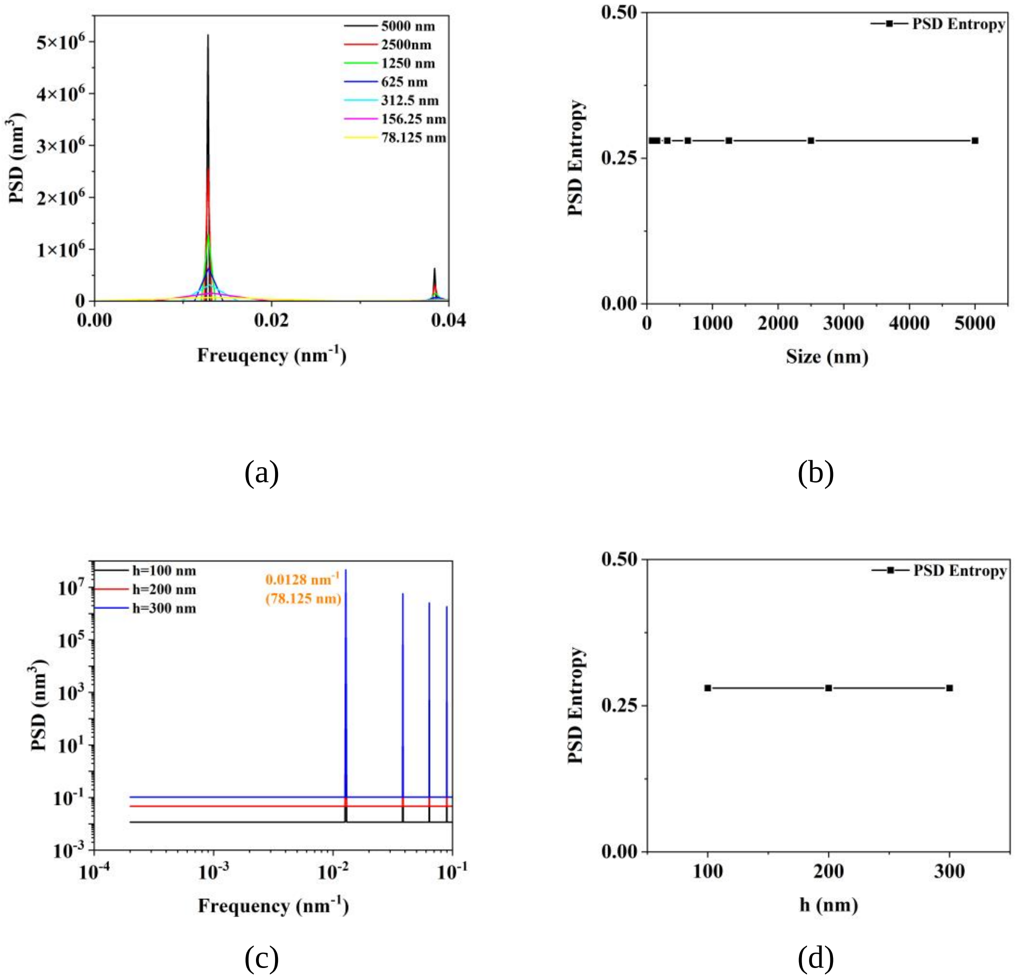

Figure S6 Simulated PSD (a) curves and (b) entropies of a diffraction grating with rectangular profiles and a period of 78.125 nm at different scanning sizes of AFM images. The scanning lengths of the corresponding AFM images are 78.125 nm, 156.25 nm, 312.5 nm, 625 nm, 1.25 μm, 2.5 μm and 5 μm. The grating height and fill factor are 100 nm and 0.5, respectively. Simulated (c) PSD curves and (d) PSD entropies of ideal patterns, i.e., diffraction gratings with rectangular profiles at different heights (h). The grating period (d) and fill factor are 78.125 nm and 0.5, respectively.

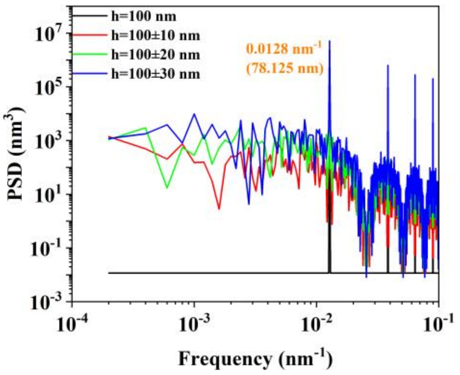

(a) Left

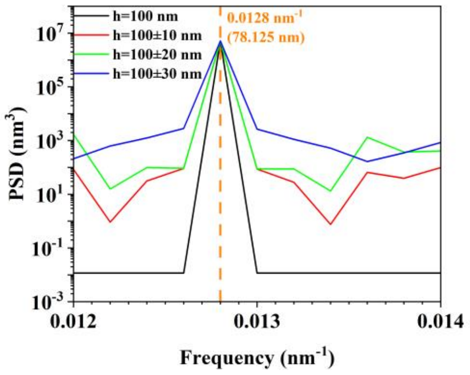

(a) Right

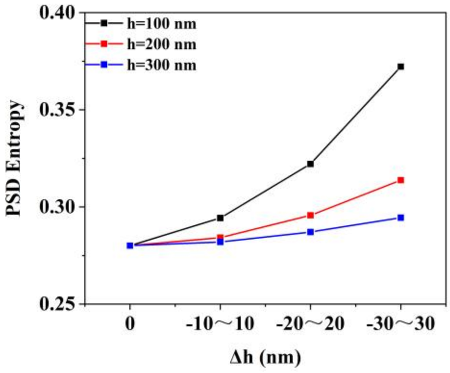

(b)

Figure S7 (a) Left: Simulated PSD curves of diffraction gratings with rectangular profiles with an average height of 100 nm at different height fluctuations Δh. Right: Simulated PSD curves for frequencies ranging from 0.012 nm^{-1} to 0.014 nm^{-1} of diffraction gratings with rectangular profiles with an average height of 100 nm, 200 nm and 300 nm at different height fluctuations Δh. The grating period (*d*) and fill factor are 78.125 nm and 0.5, respectively. (b) Simulated PSD entropies of diffraction gratings with rectangular profiles with three average heights of 100 nm, 200 nm and 300 nm at different height fluctuations Δh. The grating period (*d*) and fill factor are 78.125 nm and 0.5, respectively.

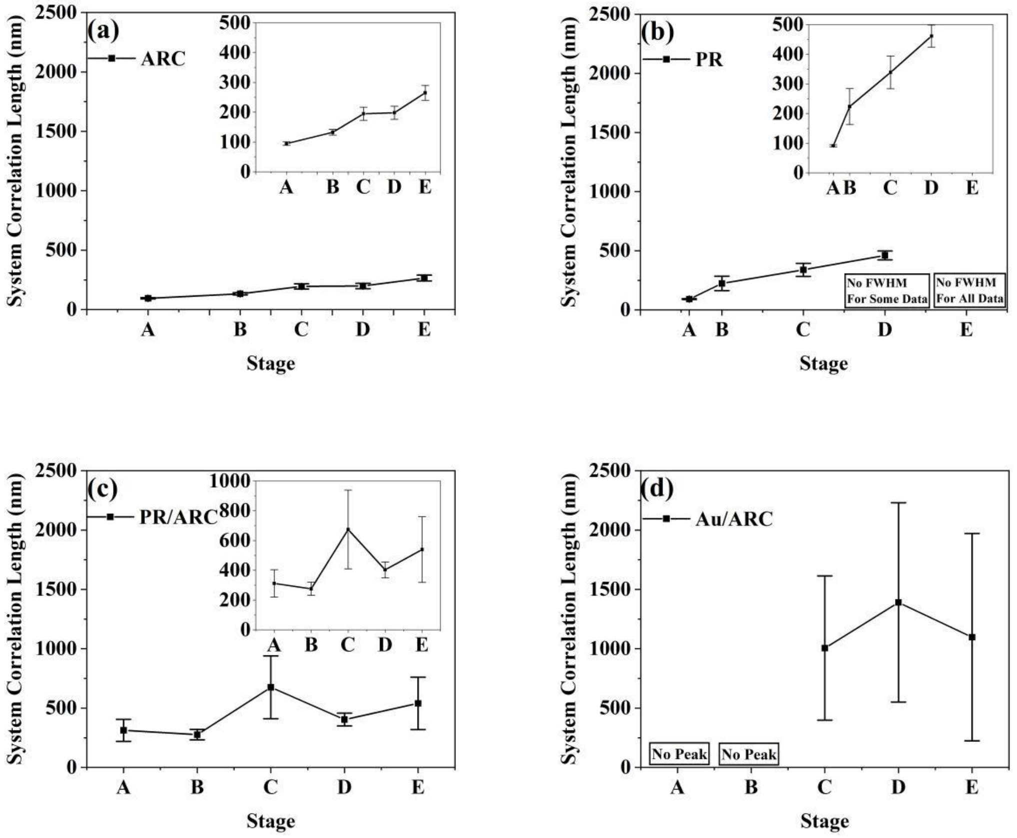

Figure S8 Lateral correlation length of the nanoripples on (a) ARC, (b) PR, (c) PR/ARC bilayer and (d) Au/ARC surfaces from the corresponding AFM morphologies for bombardment stages A–E. Inset of (a–c) shows the lateral correlation length of the nanoripples on (a) ARC, (b) PR and (c) PR/ARC bilayer with an enlarged y-axis scale.
